# Mathematical Modelling and Simulation Research of Thermal Engraving Technology Based on PMMA Material

**DOI:** 10.3390/mi7030037

**Published:** 2016-02-26

**Authors:** Xiaowei Han, Xiaowei Liu, Li Tian

**Affiliations:** Key Laboratory of Micro-systems and Micro-structures Manufacturing, Harbin Institute of Technology, Harbin 150000, China; lxw@hit.edu.cn (X.L.); tianli@hit.edu.cn (L.T.)

**Keywords:** microchannel, MEMS, microfluidic chip

## Abstract

We proposed a thermal engraving technology based on heat transfer theory and polymer rheology in microfluidic field. Then, we established a 3D model of the thermal engraving process based on polymethyl methacrylate (PMMA) material. We could employ the model to analyze the influence of temperature and speed on microchannel processing through the finite element simulation. Thus, we gained the optimal processing parameters. The orthogonal experiments were carried out within the parameter ranges obtained by the simulation results. Finally, we fabricated the smooth microchannel, the average roughness of which was 0.3 μm, by using the optimal parameters. Furthermore, we examined the surface morphology and wettability. Our work provides a convenient technological support for a fast, low-cost, and large-scale manufacturing method of microfluidic chips.

## 1. Introduction

There were many detection modes in trace detection in microfluidic field, such as photoelectric detection and capacitance coupled contactless conductivity detection in K^+^ testing [1,2]. With the development of this technology, many researchers conducted studies on the microfabrication technologies of rigid polymers. Polymer microfluidic chips were fabricated by means of silicon dry etching, electroplating and injection molding. The method could be used for fabrication of microchannels. The depth of microchannels were in the range between 100 nm and 100 μm [3]. However, this fabrication process needed direct photolithography method. Besides, microstructures could be fabricated by using hot embossing techniques in polymethyl methacrylate (PMMA) from Ni-based molding dies in 5 min. The dies were prepared by using LIGA (Lithographie, Galvanoformung, Abformung) technology [4]. The PMMA microfluidic chip could also be fabricated by means of copper master hot embossing. However, the copper master used the electroplating mold technology for forming the microstructures [5]. We found that the hot embossing method should involve indirect photolithography technology, or even combine several fabrication processes at the same time. Those methods significantly increased preparation time, cost, and complexity. The microchannel should also be processed without photolithography technologies, such as laser ablation method. The PMMA microchannels were fabricated by CO_2_ laser processing method. The two-pass laser was used to fabricate microchannel. Then, the width of microchannel was usually on the level of one hundred micrometer [6,7]. However, the size of PMMA microchannels were big when the CO_2_ laser was involved. The Nd: YAG solid-state laser was used to fabricate smaller size microchannels. The minimum average width of microchannel was 97 μm, and the minimum average depth was 36 μm [8]. Furthermore, the microchannels were fabricated by femtosecond laser technology [9]. The average surface roughness of PMMA microchannel was only 0.3 μm when the femtosecond laser was involved [10,11]. However, the femtosecond laser is usually expensive. This article proposes a thermal engraving technique applied to PMMA material and establishes the corresponding simulation models. This technique can be applied for the fabrication of microchannels in microfluidic chips without the need for photolithography. In view of the large differences of rheological mechanism between thermal engraving process and other microchannel processing methods, the modeling methods of other microchannel processing techniques are not applicable to rheological mechanism analysis of thermal engraving process. In this study, based on the rheological mechanism analysis of the thermal engraving process, a model was established. According to simulation results, reasonable experimental parameter ranges were determined, to provide technical basis for experimental studies of thermal engraving.

## 2. Design of Experimental System

**Figure 1:**
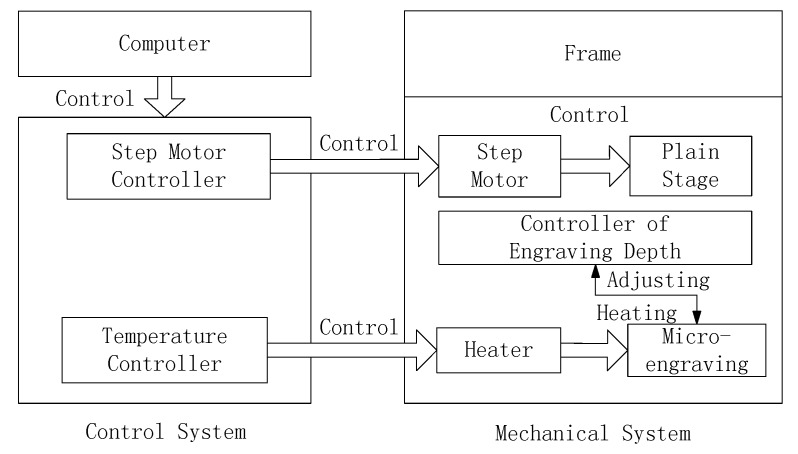
The block diagram of thermal engraving system design.

We designed and manufactured the thermal engraving system which was used to carry out the experiments. The thermal engraving system is composed of control system and mechanical system. The control system includes a temperature controller, a step motor, and an upper computer. The temperature controller and the step motor controller are used for the precise control of temperature and movement of the objective table. The mechanical system includes mainly a rack, a thermal engraving depth controller, a heater, a step motor, a micrograver, and a two-degrees-of-freedom (2-DOFs) objective table. By using the thermal engraving depth controller, the thermal engraving depth of microchannels is set. The step motor accurately controls the movement velocity of the objective table and the heater heats the micrograver. The block diagram of the thermal engraving system is shown in Figure 1. 

The temperature controller is composed of a PID (Proportion, Integration, Differentiation) controller and a K-type thermocouple. The thermocouple could convert the temperature changes to current changes and feedbacks to the PID controller. We use self-tuning function to control the temperature of micrograver during the experiment [12]. The step motor controller circuit consists of a power circuit, a step motor drive circuit and a universal serial bus (USB) drive circuit. During the thermal engraving process, the step motor controller transforms the control signal into the step motor angular displacement. Since the step motor rotates a fixed angle in each pulse single, its angular displacement can be controlled by adjusting the number of pulses. Furthermore, by varying the frequency, both the step motor rotary speed and acceleration can be adjusted. Finally, the movements of the objective table can be precisely controlled. 

**Figure 2:**
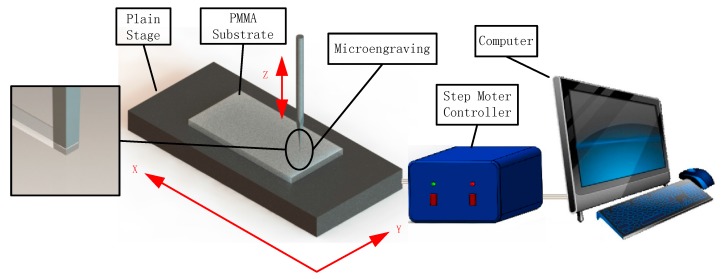
The schematic of thermal engraving process.

Figure 2 illustrates the thermal engraving process. The PMMA substrate was fixed on the objective table. The rotary speed of step motor was set through the computer software interface. Then, we set the PID controller to self-tuning mode. The temperature of the micrograver would reach the setting value after the self-tuning process. Therefore, we can carry out the thermal engraving experiments.

## 3. Modeling, Simulation and Result Analysis

Using the established micrograver heat transfer model, the temperature gradient of the micrograver at different temperatures was simulated. The simulation results can provide the theoretical basis for analyzing the heat transfer characteristics of the micrograver and PMMA material. Moreover, based on the simulation results the PMMA viscous flow range around the micrograver can be determined, and the geometric model of PMMA viscous flow built. By analyzing the forces applied on the PMMA viscous flow under the conditions with varying technological parameters, a reasonable range for technological parameters in thermal engraving experiments can be concluded.

### 3.1. Thermal Micorgraver Head Heat Transfer Analysis

Since the temperature controller maintains the micrograver at a preset temperature, we mainly focused on the steady-state heat transfer model of the micrograver. One assumes that during the modeling process the heater heat completely transfers to the micrograver. The contact area between the micrograver and the air far exceeds the contact area between the micrograver and the PMMA material; therefore, only the thermal transfer between micrograver and air was modeled, neglecting the effects induced by heat transfer between micrograver and PMMA material. Finally, when simulating the micrograver temperature distribution, the air temperature was set to room temperature.

#### 3.1.1. Geometric Model

**Figure 3:**
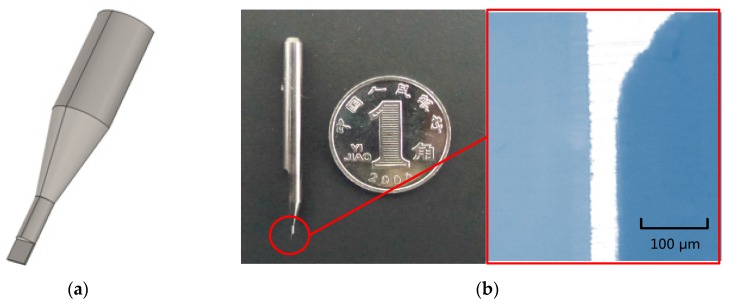
The geometric model and the photo of a rectangular engraving micrograver: (**a**) The geometric model of a rectangular engraving micrograver; (**b**) The photo of a rectangular engraving micrograver.

Figure 3 displays the geometric model and the photo of a rectangular micrograver. Figure 3b is a photo of the minimum rectangular micrograver which we could fabricate. To reduce the computational load, only the connecting part between micrograver and heater has been modeled. Therefore, the temperature at the cross section of micrograver is the heater heating temperature.

The micrograver main body is a tungsten steel bar whose diameter is 3.75 mm. The characteristic size (width) of the micrograver head is 100 μm; thus, a large geometric size span exists. Therefore, a reasonable finite element mesh generation of the geometrical model improves the simulation precision.

#### 3.1.2. Micrograver Head Heat Transfer Analysis

**Figure 4:**
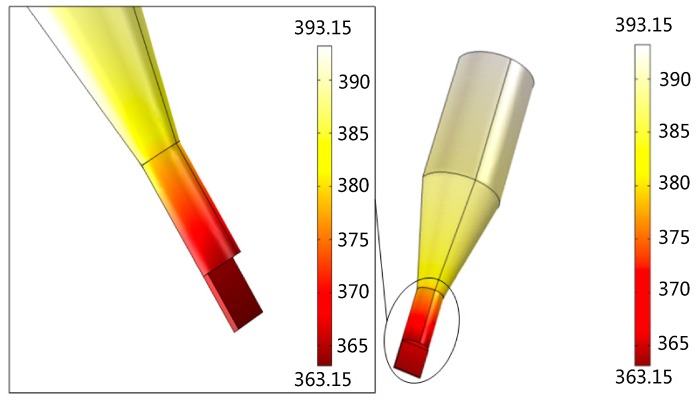
The surface temperature distribution of the thermal engraving micrograver.

Figure 4 displays the surface temperature distribution of thermal micrograver. When the temperature of the heating zone is 120 °C, the micrograver head temperature is only 90 °C, *i.e.*, with a temperature difference of 30 °C. As shown in the partial enlarged view of the surface temperature distribution of the micrograver head, the geometrical size decreases rapidly while the ratio of surface to volume increases. However, since the micrograver head length is only 2 mm, its temperature gradient exhibits no obvious increase.

**Figure 5:**
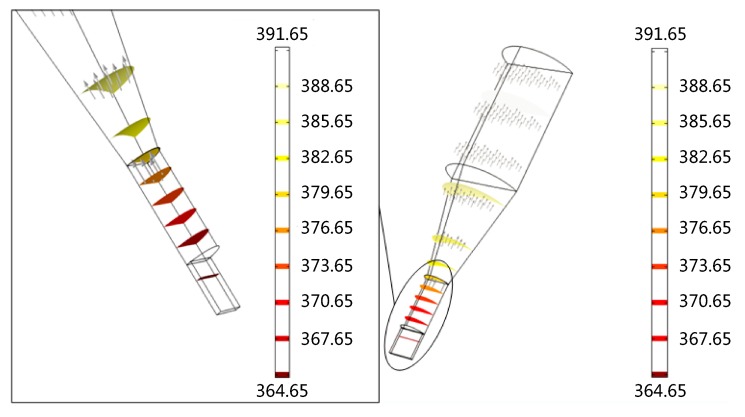
The cross sectional view of temperature distribution of the thermal engraving micrograver.

Several cross sections were selected along the axial direction of thermal micrograver for further analyzing its heat transfer behavior. As Figure 5 shows, when the temperature of the heating zone is 120 °C the temperature gradient in the micrograver head is less than 3 °C. Due the difference between the size of the micrograver head length and the depth of thermal engraving, the temperature gradient of the contact area between the micro-engraver and PMMA can be neglected.

### 3.2. Temperature Distribution of PMMA Materials

**Figure 6:**
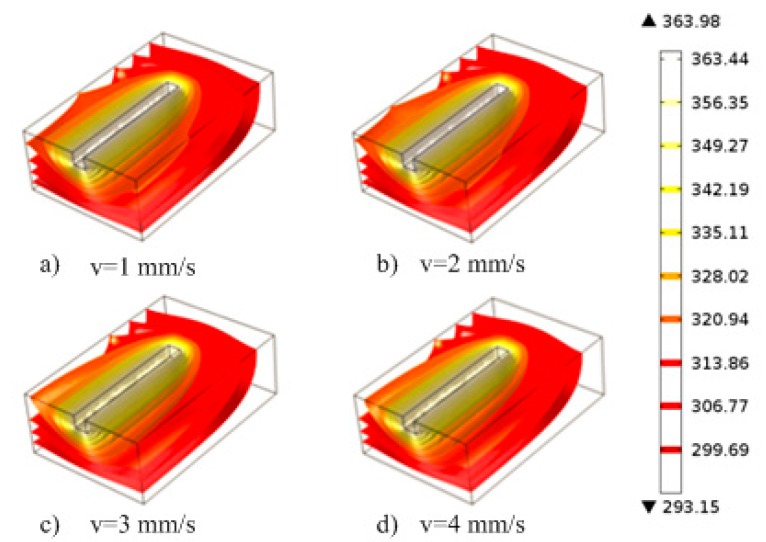
The cross-sectional view of temperature distribution at different thermal engraving speed: (**a**) *v* = 1 mm/s; (**b**) *v* = 2 mm/s. (**c**) *v* = 3 mm/s; (**d**) *v* = 4 mm/s.

During thermal engraving process, when the temperature parameter is fixed, the thermal engraving velocity can influence the material temperature distribution. Figure 6 shows the temperature distributions of PMMA materials when the temperature parameter is 91 °C, and the thermal engraving velocities are 1 mm/s, 2 mm/s, 3 mm/s and 4 mm/s, respectively. The viscous transition temperature of PMMA materials was 89 °C. At 91 °C, the viscous flow range of PMMA near the micrograver head is quite small; at temperatures less than 89 °C, PMMA materials around the micrograver head become non-viscous flow. When the applied force exceeds the elastic limit, fractures may occur in non-viscous PMMA materials, thus it is difficult to fabricate microchannels with smooth surface morphology. Therefore, the thermal engraving velocity should be matched with heat transfer velocity, to keep PMMA near the micrograver always in the viscous flow state. Moreover, as the thermal engraving velocity increases, the isothermal surface of PMMA near the engraver head shrinks gradually towards the micrograver head.

**Figure 7:**
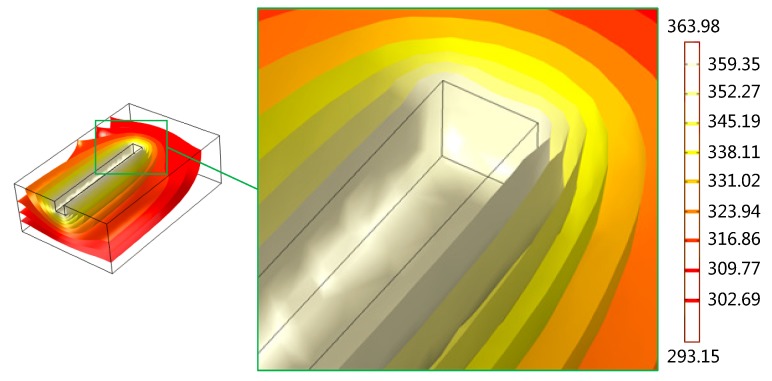
The cross-sectional view of temperature distribution when the thermal engraving speed is higher than the heat transfer rate.

When the thermal engraving velocity increases to a certain degree, the micrograver head velocity will exceed the heat transfer velocity, *i.e*., the thermal engraving velocity may not match the temperature. At this moment, there will exists insufficient PMMA viscous flow layer along the movement direction of micrograver head, and the micrograver head will directly contact the elastomeric-state or glassy-state PMMA materials. Figure 7 shows the temperature distribution of PMMA material when the temperature is 91 °C and the thermal engraving velocity is 5 mm/s. PMMA materials within the range of 10 μm around the micrograver head are all in non-viscous flow state when the temperature is 96 °C. Consequently, at 91 °C, the thermal engraving velocity should not exceed the upper limit of 5 mm/s. When the velocity exceeds this limit, it became hard to fabricate micro-channels with smooth surface topography. When the temperature increases, since the temperature gradients of the micrograver head and PMMA increase, a higher upper limit of thermal engraving velocity is allowed.

### 3.3. Thermal Engraving Model

During the modeling of the heat transfer between micrograver head and PMMA viscous flow, the material specific heat capacity and thermal conductivity are relatively low and the viscous flow steadily extrudes. The heat exchange between the extruded PMMA viscous flow and air during the dynamical thermal engraving process is thus neglected. Since the temperature controller keeps the micrograver head temperature at a constant value, the heat exchange with PMMA material does not reduce the surface temperature of micrograver head. Moreover, when simulating the heat transfer model between micrograver head and PMMA material the initial temperature of PMMA was set to room temperature (20 °C). 

#### 3.3.1. Construction of the Geometric Model of the Viscous Flow Microchannels

**Figure 8:**
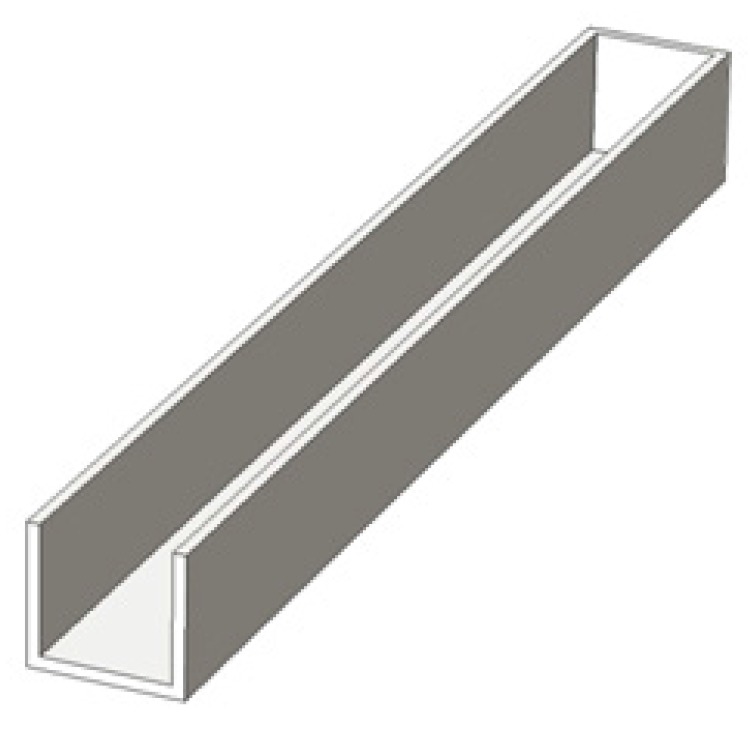
The geometrical model a 100 μm width rectangular microchannel in the thermal engraving process.

Figure 8 shows the microchannel geometrical model during thermal engraving process when a rectangular thermal micrograver with both depth and width of 100 μm was used. To highlight viscous flow movements into substrate, the micrograver head was modeled transparent; the outside surface of the head was set as the internal geometric boundary. Besides, the extruded viscous flow is irrelevant in micro-channel processing and therefore the front viscous flow of PMMA has not been modeled.

#### 3.3.2. Temperature Field Distribution in the Viscous Flow Channel

**Figure 9:**
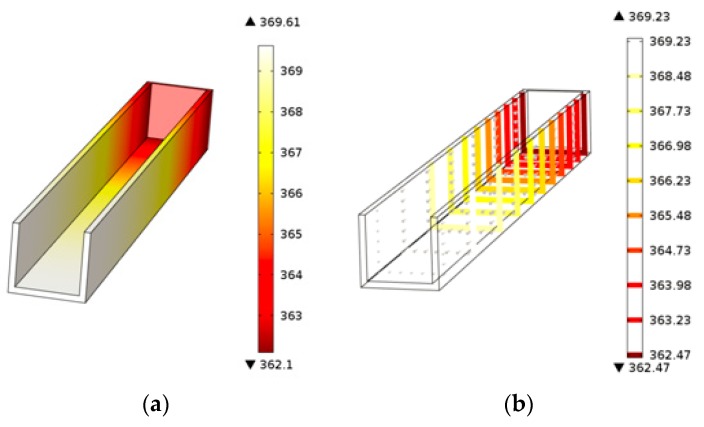
The temperature of the polymethyl methacrylate (PMMA) viscous flow: (**a**) The temperature distribution of the PMMA viscous flow; (**b**) The isothermal sectional view of PMMA viscous flow.

Figure 9a displays the temperature distribution of the PMMA viscous flow during the thermal engraving process when the micrograver head temperature is 96 °C and the velocity is 3.2 mm/s. Since the PMMA viscous flow was constantly extruded along the micrograver head movement direction, the temperature of the micrograver head is obviously lower than that of the PMMA viscous flow on its both sides. Figure 9b shows the isothermal profile of the PMMA viscous flow during the engraving process. The distance between two isothermal surfaces of PMMA viscous flow below the micrograver head is relatively large. This means that the temperature gradient of the viscous flow on the bottom of the head decreases gradually along the vertical direction because the heat conductivity coefficient and specific heat capacity of PMMA viscous flow are small, and thereby the heat transfer inside the viscous flow is quite slow. Besides, since the micrograver is in motion, the head-on heat flux along the micrograver movement direction is larger. This leads to a greater temperature gradient of PMMA viscous flow in front of the micrograver head while the temperature gradient along the normal direction of the micrograver movement is smaller. Meanwhile, the temperature in front of the micrograver is much lower than those on two sides and on bottom of the micrograver head.

#### 3.3.3. Viscous Flow Field Distribution

**Figure 10:**
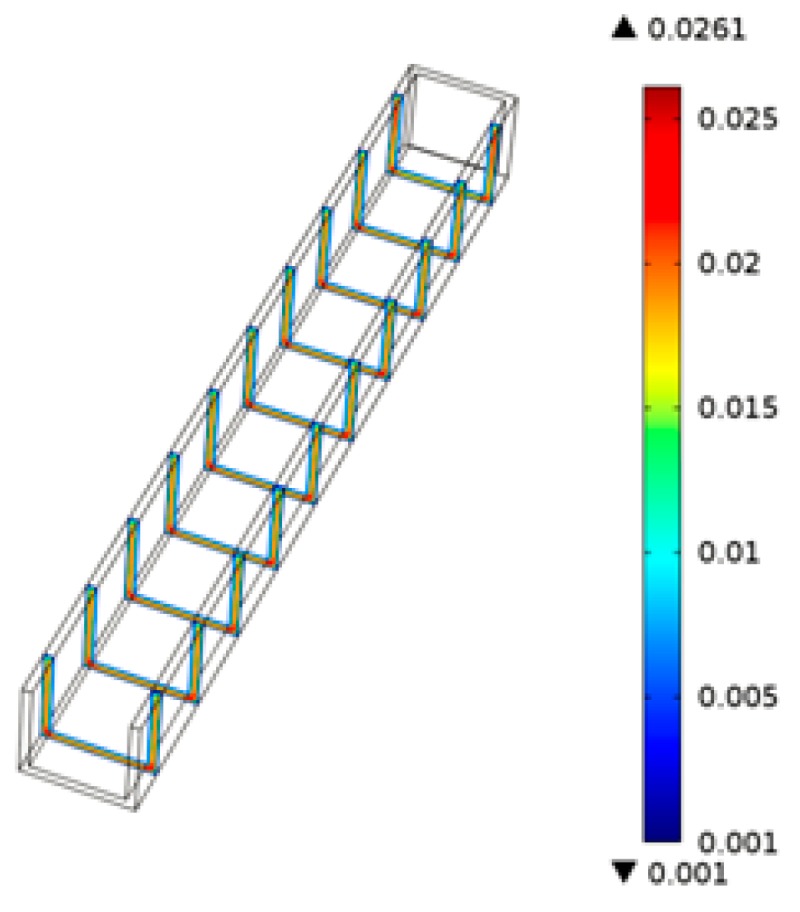
The flow field of the PMMA viscous flow.

Figure 10 shows the flow field distribution of the PMMA viscous flow around the micrograver head during thermal engraving process. The flow velocity of the PMMA viscous flow around the micrograver is relatively large. As the thickness of viscous flow increases, the flow velocity near the boundary of viscous flow microchannel decreases gradually.

#### 3.3.4. Viscous Flow Pressure Distribution

**Figure 11:**
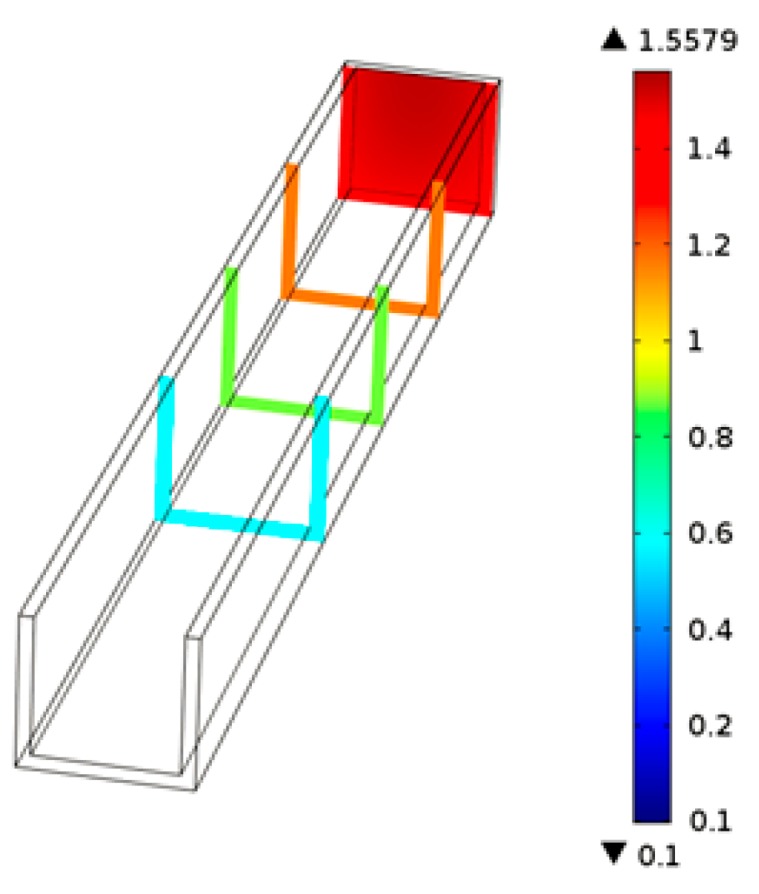
The cross-sectional view of the pressure distribution of the PMMA viscous flow.

Under the effect of thermal micrograver movements, the head-on PMMA viscous flow in the micrograver head movement direction would be squeezed by micrograver head. Figure 11 shows the pressure distribution profile of the PMMA viscous flow: the pressure on the head-on viscous flow in the micrograver head movement direction is the largest and decreases gradually along the microchannel (in a backwards direction).

#### 3.3.5. Viscous Flow Microchannel Pressure Distribution

**Figure 12:**
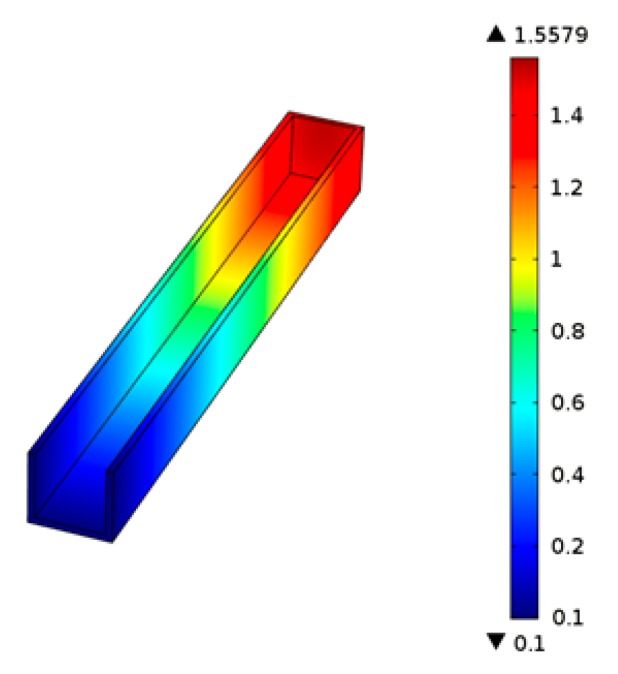
The pressure distribution of the PMMA viscous flow.

The PMMA viscous flow stress analysis gives its pressure distribution. According to Figure 12, when the PMMA viscous flow extrudes from the front of micrograver head, the pressure on the head-on viscous flow in the micrograver head movement direction is the largest and decreases gradually backwards. Since some viscous flow extrudes from the top, the pressure at the exit of the viscous flow decreases slightly.

### 3.4. Result Analysis

The thermal engraving model presents the 3D results of temperature distribution, flow distribution, viscous field stress distribution, and boundary pressure distribution of PMMA viscous flow under specific technological parameters. However, the simulation analyses using a single group of technological parameters can hardly offer a clear and regular understanding of the thermal engraving process or determine a reasonable technological parameters range. Therefore, the simulation results of the PMMA viscous flow under different parameters have been systematically analyzed, to obtain the trend of simulation results.

#### 3.4.1. Effects of the Temperature

**Figure 13:**
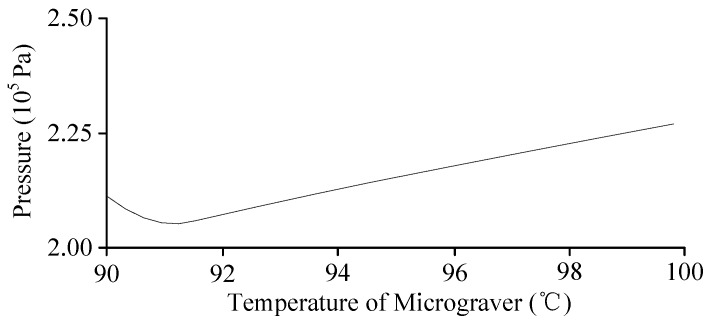
The relationship between temperature and pressure of viscous flow on the normal movement direction of micrograver.

With the same thermal engraving velocity, different temperatures would impose a certain effect on the viscosity of the flow and then affect the pressure distribution of the viscous flow. When the stress was applied on the viscous flow through the micrograver head, the generated pressure affected the microchannel fabrication. Figure 13 shows the variation of pressure on the viscous flow with temperature along the normal direction of the micrograver head movements.

#### 3.4.2. Effects of the Speed

**Figure 14:**
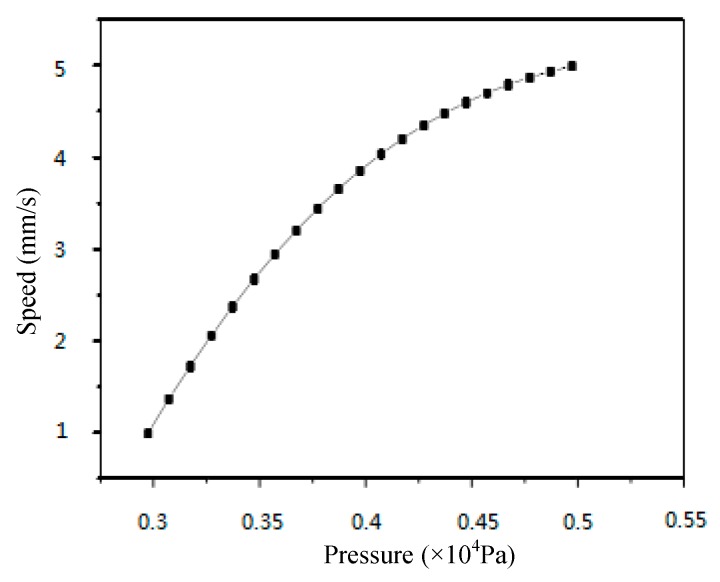
The relationship between the speed and the pressure of viscous flow on the normal direction of micrograver.

Changes in velocity can lead to the variation of pressure on the PMMA viscous flow around the micrograver head. Figure 14 shows the pressure on the PMMA viscous flow when the micrograver head velocity exhibits a normal spatial relation with its movement direction.

## 4. Microchannel Characterization

### 4.1. Surface Topography Characterization

**Figure 15:**
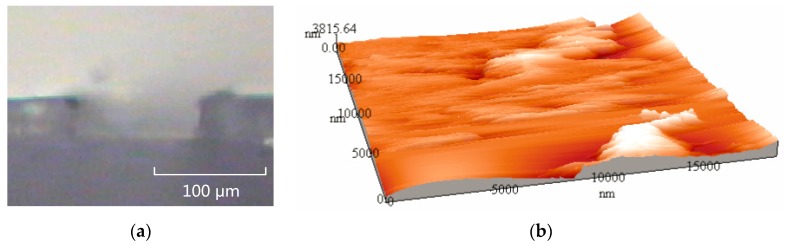
The features of rectangular microchannel: (**a**) The cross-sectional image of rectangular microchannel; (**b**) The atomic force microscope (AFM) image of microchannel.

Orthogonal tests were performed using the parameters determined by numerical simulations, and finally the optimal technological parameters were acquired. When the thermal engraving velocity is 4.2 mm/s and the temperature is 91 °C, the 0.3 μm rough microchannels can be fabricated by thermal engraving depth ranging from 30 to 200 μm. The thickness deviation of PMMA plant we involved was between 10 and 20 μm. So, we couldn’t guarantee the fabrication quality if the thermal engraving depth was below 30 μm. Figure 15a illustrates a cross-sectional image of rectangular microchannel. Figure 15b shows the atomic force microscope (AFM) image of microchannel bottom surface. 

**Figure 16:**
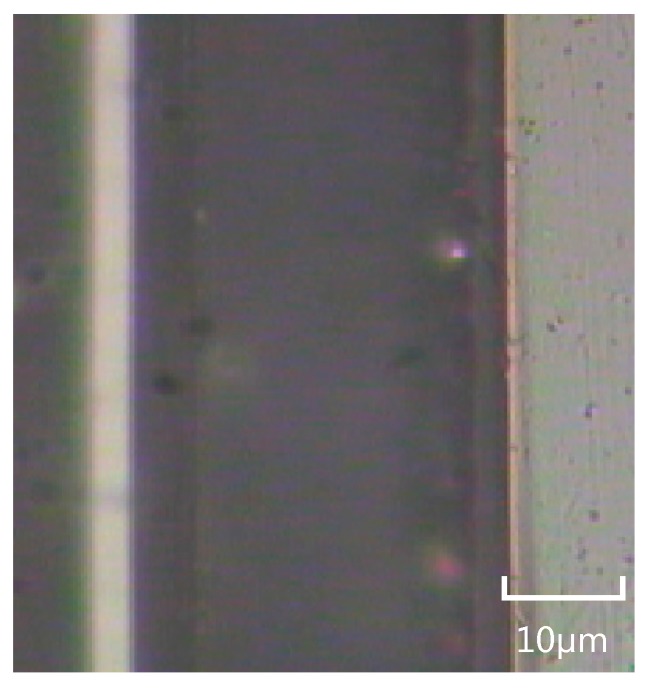
The 30 μm width rectangular microchannel.

Figure 16 shows a 30 μm width rectangular micro-channel; its size can be comparable to the size of microchannels fabricated by means of lithography or laser techniques [13,14]. 

We supply the comparison as Table 1. In this table, we compare our method with some representative methods, such as laser ablation method, hot embossing method and so on. 

We find the thermal engraving method gain some advantages microchannel size, process cycle and cost. Moreover, this method don’t involve any assistant technology, such as electrical discharge machining (EDM), physical vapor deposition (PVD) and ultraviolet lithography (UV Lithography). 

### 4.2. Wettability Characterization

During thermal engraving, temperature and velocity may affect the microchannel wettability to a certain degree. The wettability can then affect the flow resistance of the solution in the microchannel. Therefore, in the present work heating temperature of the micrograver head and microchannel surface roughness characterize microchannel wettability.

#### 4.2.1. Effects of Temperature on Microchannel Surface Wettability

Surface free energy γ_sv_ (*i.e.*, surface tension) and surface roughness are two primary factors usually affecting wettability. However, for solid materials, γ_sv_ measurement is a difficult task. Thus, we use the contact angle between liquid and PMMA surface to assess the variation of the solid surface energy. Therefore, we designed a set of experiments to assess the effects of the thermal engraving temperature on the surface energy of PMMA material. So, we could studied the effects of thermal engraving temperature on micro-channel wettability.

The PMMA substrate was only heated by vacuum drying oven without any engraving and the variation of contact angle directly reflects the effect of temperature variation on wettability of PMMA materials. The substrate was heated within the range 89 °C to 95 °C. Experimental data are show in Table 2. The larger contact angle means poorer hydrophilicity. We found the hydrophilic decreased with the increase of temperature. The experimental data also show the change of water contact angle is 4.8% in the temperature range of 89–90 °C. So, we could deduce the effects of temperature parameter on the hydrophilicity is limited.

#### 4.2.2. Effects of Roughness on Microchannel Surface Wettability

**Figure 17:**
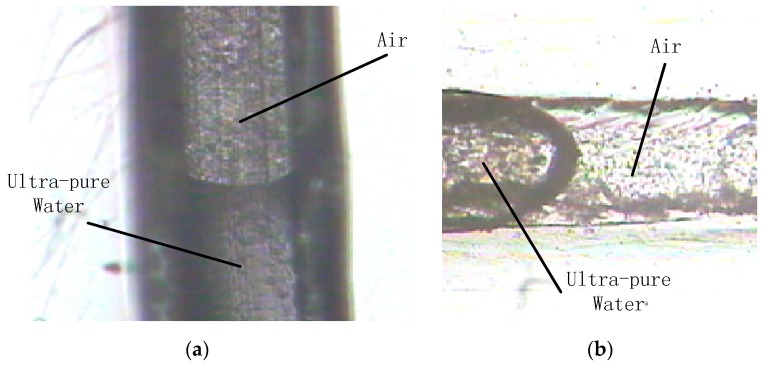
The wettability of microchannels: (**a**) Hydrophilic microchannel; (**b**) Hydrophobic microchannel.

At the gas-liquid interface, the microchannel wettability can be evaluated by observing the flow pattern in front of the liquid. The liquid flowing head, convex or concave, would indeed be influenced by the pressure of the flowing. We did not bond the microchannel in the process of hydrophilic test. Thus, we couldn’t apply any pressure since we considered the pressure’s influence. It is a kind of natural flowing without any external force applied to the liquid flowing. Figure 17a shows a flat and smooth microchannel using the optimal technological parameters. At the gas-liquid interface the meniscus can be observed, *i.e*., the microchannel is hydrophilic. By way of comparison, Figure 17b shows a rough microchannel using non-ideal technological parameters. There is so meniscus at the gas-liquid interface, *i.e*., the microchannel is hydrophobic.

## 5. Conclusions

In this paper, a thermal engraving microchannel fabrication technique without the application of lithography was proposed and the related manufacturing process model was constructed. In the model, the stresses on the viscous flow around the micrograver head using different technological parameters were analyzed and the reasonable range of technological parameters was then determined. Results show that, when the temperature ranges from 90 to 95 °C, the stress on the viscous flow around the micrograver head is relatively small. Moreover, as the velocity increases, the pressure on the normal direction of the micrograver movements increases nonlinear. Finally, orthogonal experiments were conducted using the selected optimal technological parameters, and a microchannel with the roughness and minimum characteristic sizes of 0.3 and 30 μm, respectively was fabricated. The experimental results indicate that when the microchannel was fabricated under the condition with optimal technological parameters has flat and smooth surface, and thus is hydrophilic; on the contrary, under non-ideal parameters is characterized by rough surface and hydrophobicity. The presented results are applicable to the analytical chemistry laboratory with no superclean room and expensive lithography equipment, which can also be further extended to the micro-channels fabrication in non-crystalline and linear polymers applicable to microfluidic fields.

## Figures and Tables

**Table 1:** The comparison of microchannel fabricating methods based on PMMA.

Method	Microchannel Size (μm)	Surface Roughness (μm)	Process Cycle (min)	Assistant Technology	Cost	Literature
Injection moulding	48 × 110	<0.001	-	EDM, PVD	High	[15]
CO_2_ laser ablation	150 × 50	25	<1	None	Low	[7]
Femtosecond laser ablation	100 × 20	0.05–0.3	<1	Chloroform vapor Treatment	Low	[11]
PCB mold hot embossing	400 × 25	0.56 ± 0.13	5	UV Lithography	Low	[16]
SU-8 mold hot embossing	15 × 50	0.7	60–80	UV Lithography	High	[17]
Thermal engraving	30 × 30	0.3	<1	None	Lower	This paper

**Table 2:** The relationship between heating temperature and contact angle.

**Temperature (°C)**	89	90	91	92	93	94	95
**Contact angle (°)**	77.4	77.8	78.3	79.4	79.9	80.6	81.1
